# Contamination and Health Risk Assessment of Potentially Toxic Elements in Household Dust Across the Haze Season in Upper Northern Thailand

**DOI:** 10.3390/toxics13121008

**Published:** 2025-11-21

**Authors:** Kawinwut Somsunun, Teetawat Santijitpakdee, Kanyapak Kohsuwan, Natwasan Jeytawan, Sukrit Kirtsaeng, Dan Norbäck, Tippawan Prapamontol

**Affiliations:** 1 Environment and Health Research Group, Research Institute for Health Sciences (RIHES), Chiang Mai University, Chiang Mai 50200, Thailand; kawinwut.som@cmu.ac.th (K.S.); santijitpakdee.t@gmail.com (T.S.); kanyapak_koh@cmu.ac.th (K.K.); natwasan_jeytawan@hotmail.com (N.J.); 2 Office of Research Administration, Chiang Mai University, Chiang Mai 50200, Thailand; 3 Northern Meteorological Center, Meteorological Department, Chiang Mai 50200, Thailand; sukritk@hotmail.com; 4 Department of Medical Sciences, Uppsala University, 75185 Uppsala, Sweden; dan.norback@medsci.uu.se

**Keywords:** potentially toxic elements, indoor household dust, haze season, health risk assessments, upper northern Thailand

## Abstract

Indoor exposure to potentially toxic elements (PTEs) presents a global health concern, yet comprehensive seasonal assessments in Thailand remain limited, particularly during air pollution episodes. We assessed 15 PTEs in household dust collected across eight provinces of upper northern Thailand (UNT) during the haze and non-haze seasons to evaluate contamination levels, identify sources, and assess health risks. Five elements (Cr, Mo, Ni, Pb, and Zn) showed significantly higher concentrations during the haze season (*p* < 0.05), accompanied by corresponding increases in contamination indices and more diverse pollution sources being identified compared to the non-haze season, with Sb showing the highest enrichment degree (EF = 117.8). Source identification revealed potential enrichment from mixed anthropogenic sources, natural soil, industrial activities, agricultural inputs, and biomass burning. Health risk assessment showed that children faced unacceptable non-carcinogenic risks (HI = 2.51), increasing to 2.79 during the haze season, exceeding safe thresholds. Both adults and children experienced unacceptable carcinogenic risks from chromium exposure, particularly through inhalation during haze episodes. Total lifetime cancer risks increased from 1.20 × 10^−4^ to 1.74 × 10^−4^ for children and from 4.02 × 10^−4^ to 6.06 × 10^−4^ for adults during the haze season. These findings underscore the critical need for integrated pollution control strategies addressing biomass burning emissions to reduce indoor dust contamination and protect public health in biomass-burning-impacted regions.

## 1. Introduction

People worldwide spend approximately 80–90% of their time indoors [1,2], making indoor environments a crucial exposure pathway for environmental pollutants. This prolonged indoor exposure is particularly concerning for children, the elderly, and vulnerable populations who spend most of their lifetime in residential settings [3]. The health burden of indoor air pollution is substantial. In 2020, household air pollution exposure was responsible for an estimated 3.2 million deaths annually, contributing to non-communicable diseases and approximately 6% of all lung cancer deaths [4]. These findings underscore the importance of identifying, characterizing, and mitigating risks associated with household contaminants.

Household dust serves as a significant reservoir for various contaminants, including potentially toxic elements (PTEs), metalloids, and organic pollutants. These contaminants can be resuspended into the air through wind and human activities, affecting both air quality and human health [5,6,7]. PTEs in dust readily transfer to humans through three primary exposure pathways including ingestion, inhalation, and dermal absorption [5,8]. Contamination in household dust originates from both internal and external sources, including transportation, fossil fuel and biomass combustion, painting, agricultural activities, and natural geological sources [5,9].

Ambient PM_2.5_ represents an important source of PTEs that correlate strongly with household dust contamination. Recent studies have demonstrated quantitative indoor-outdoor relationships for PM_2.5_ and associated pollutants in residential indoor environments [10,11,12]. PM_2.5_-bound PTEs accumulate progressively in settled dust, with infiltration rates varying by building characteristics and ventilation patterns [13,14]. This outdoor PM_2.5_ serves as a primary vector for PTE transport into indoor environments, particularly in regions with high ambient concentrations.

Upper northern Thailand (UNT) experiences severe air pollution episodes during dry seasons when biomass burning from agricultural activities and forest fires generate the extremely high PM_2.5_ concentrations [15,16]. These haze events substantially elevate atmospheric concentrations of fine particulate matter and associated PTEs [16,17]. Biomass-burning emissions contain elevated PTE concentrations that can infiltrate indoor environments and accumulate in household dust [13,18]. Despite the severity and regularity of these pollution episodes, comprehensive assessments of PTE contamination in indoor household dust across both haze and non-haze seasons remain limited. The open-air architectural design prevalent in northern Thai households facilitates outdoor pollutant infiltration, potentially increasing PTE deposition in household dust during pollution episodes [12,19]. The mechanisms by which outdoor particulate matter infiltrates indoor environments through natural ventilation have been well documented, establishing a direct link between ambient air quality and indoor dust contamination [13,14]. This relationship becomes particularly pronounced in regions experiencing severe seasonal air pollution.

Previous studies have documented elevated PTE concentrations in household dust globally, with sources ranging from anthropogenic activities to natural soil resuspension [5,9]. In Thailand, limited research has examined indoor dust contamination, primarily focusing on urban areas or specific activities and identifying mixed PTE sources [20,21]. However, critical knowledge gaps limit our understanding of biomass-burning impacts on household dust contamination. While comprehensive multi-provincial assessments comparing seasonal variations remain scarce, the challenges extend beyond temporal analysis. Systematic evaluation of PTE contamination patterns across pollution and non-pollution periods, quantitative source apportionment to distinguish biomass burning from other anthropogenic and indoor sources, comparative health risk assessment between seasonal conditions, and understanding of pollutant infiltration dynamics in tropical open-air architecture all require further investigation. These interconnected gaps hinder the development of evidence-based pollution control strategies and targeted public health policies for biomass-burning-impacted regions.

This study comprehensively assessed contamination levels and health risks associated with 15 PTEs including aluminum (Al), arsenic (As), boron (B), barium (Ba), cadmium (Cd), cobalt (Co), chromium (Cr), copper (Cu), manganese (Mn), molybdenum (Mo), nickel (Ni), lead (Pb), antimony (Sb), vanadium (V), and zinc (Zn) in indoor household dust across eight provinces in UNT during both haze and non-haze seasons. Specific objectives included: (1) quantifying PTE concentrations and comparing levels between seasonal episodes; (2) evaluating contamination status using multiple pollution indices, including Enrichment Factor (EF), Contamination Factor (CF), Pollution Load Index (PLI), and Index of Geo-accumulation (Igeo); (3) identifying potential PTE sources through principal component analysis; and (4) assessing both non-carcinogenic and carcinogenic health risks for children and adults through ingestion, inhalation, and dermal contact pathways using US EPA risk assessment models. This research provides critical insights into seasonal variations in indoor dust contamination patterns and associated health risks in a region severely affected by transboundary haze pollution. The findings will support the development of evidence-based pollution control strategies and public health policies for indoor environmental quality management in biomass-burning-impacted regions.

## 2. Materials and Methods

### 2.1. Study Area

**Figure 1:**
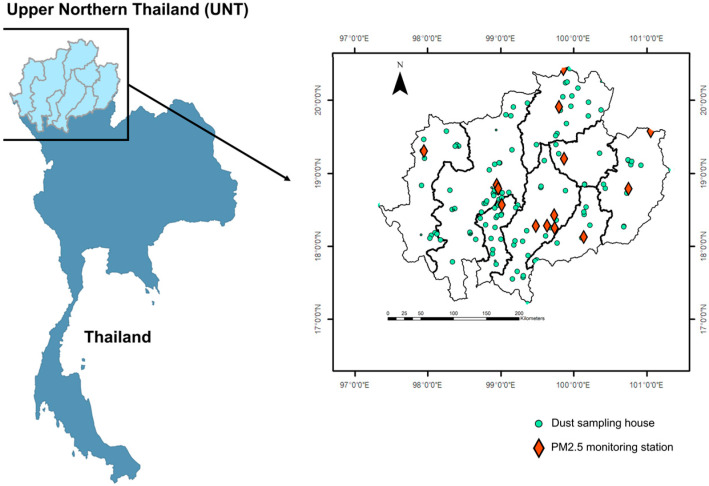
Study area in eight provinces of upper northern Thailand (UNT) with sampling location of household dust collection (the green points) and PM_2·5_ monitoring station (the red diamonds).

This study encompassed eight provinces in upper northern Thailand (UNT) (Figure 1) including Chiang Mai, Chiang Rai, Lamphun, Lampang, Mae Hong Son, Nan, Phayao, and Phrae. During the dry season (February–May), the region experiences severe air pollution with 24-h average PM_2.5_ concentrations exceeding 300 µg m^−3^, 20 fold higher than WHO guidelines [16]. We selected UNT as our study area to assess health risks from PTE exposure in household dust across varying pollution seasons. Sampling locations and the study area map are presented in Figure 1.

### 2.2. Household Dust Samples and Data Collection

Between 2019 and 2020, we recruited households across eight UNT provinces with informed consent (Human Experimentation Committee, Research Institute for Health Sciences, Project No. 1/59, approved on 10 May 2016). We collected 210 indoor dust samples during both the haze and non-haze seasons. The haze seasons were defined as more than 4 consecutive days with 24-h average PM_2.5_ exceeding 15 µg m^−3^ (WHO reference level), ending when concentrations remained below this threshold for similar duration. The non-haze seasons fell below this criterion. Dust samples were vacuumed (HITACHI CV-SF18 220C) from non-ground surfaces at least 1 m above floor level, specifically including windowsills, shelves, furniture tops (tables, cabinets, wardrobes) and ceiling fans in living rooms and bedrooms. For each household, approximately 2–4 m^2^ of surface area was vacuumed over a 15–20 min sampling period. Collected dust was immediately transferred to clean polyethylene bags. Samples were then desiccated, sieved through 63-µm mesh, and stored (0.5–3 g aliquots) in sterile polyethylene bags at −20 °C until analysis.

### 2.3. Elemental Analysis

We quantified 15 PTEs (Al, As, B, Ba, Cd, Co, Cr, Cu, Mn, Mo, Ni, Pb, Sb, V, and Zn) selected based on their established health impacts and US EPA reference doses [22,23], following modified protocols [20,24]. Briefly, 150 mg of dust were digested in 5 mL HNO_3_ and 5 mL HCl using microwave digestion (ETHOS UP, Milestone Inc., Sorisole, Italy) at 210 °C for 40 min. Digests were diluted to 25 mL with Milli-Q water (18.2 MΩ-cm) and filtered (0.2 µm nylon membrane). Elemental concentrations were measured in triplicate via Inductively Coupled Plasma Optical Emission Spectroscopy (ICP-OES; Agilent 5800, Santa Clara, CA, USA). Calibration curves using certified multi-element standards (CPA Chem, Bogomilovo, Bulgaria) yielded excellent linearity (r^2^ > 0.999). Method accuracy was verified with standard reference material (SRM 2584), achieving recoveries of 80.7–107.1%. Quality control parameters, including detection limits and recoveries, are detailed in Table S1.

### 2.4. Dust Pollution Assessment

To assess the PTE contamination in household dust, we used four indices: Enrichment Factor (EF), Contamination Factor (CF), Pollution Load Index (PLI), and Index of Geo-accumulation (Igeo). These indices quantify contamination extent and identify potential sources.

#### 2.4.1. Enrichment Factor (EF)

Enrichment factor (EF) distinguishes natural from anthropogenic sources by normalizing element concentrations to a geochemically stable reference element [5,25]. The EF was calculated following Equation (1):

(1) $$EF=\frac{{\left(\frac{C_{x}}{C_{\mathrm{ref}}}\right)}_{\mathrm{sample}}}{{\left(\frac{C_{x}}{C_{\mathrm{ref}}}\right)}_{\mathrm{background}}}$$

where *C_x_* represents measured element concentrations, and *C_ref_* denotes the reference element concentration (aluminum). Background values were derived from Taylor and McLennan’s upper continental crust data [26,27]. EF values indicate enrichment levels: EF < 2 (no enrichment, natural sources), 2 ≤ EF < 5 (minor enrichment), 5 ≤ EF < 20 (moderate enrichment), 20 ≤ EF < 40 (significant enrichment), and EF ≥ 40 (very high enrichment from industrial or urban sources) [28].

#### 2.4.2. Contamination Factor (CF)

Contamination factor (CF) quantifies contamination degree by comparing measured element concentrations to geochemical background values [29]. CF was calculated as Equation (2):

(2) $$CF=\frac{C_{\mathrm{sample}}}{C_{\mathrm{background}}}$$

where *C_sample_* represents measured element concentrations and *C_background_* denotes upper continental crust values [26,27]. CF values indicate contamination severity: CF < 1 (low), 1 ≤ CF < 3 (moderate), 3 ≤ CF < 6 (considerable), and CF ≥ 6 (very high) [30].

#### 2.4.3. Pollution Load Index (PLI)

Overall pollution status was assessed using Pollution Load Index (PLI), which integrates CF values across multiple elements following Equation (3) [29]:

(3) $$PLI\;=\;{\left({\mathrm{CF}}_{1}\;\times \;{\mathrm{CF}}_{2}\;\times \;{\mathrm{CF}}_{3}\;\times \;\ldots \;\times \;{\mathrm{CF}}_{n}\right)}^{\frac{1}{n}}$$

where *n* is the number of analyzed elements, and CF presents the contamination factor for each element. PLI values indicate pollution severity: PLI < 1 (no pollution), 1 ≤ PLI < 2 (moderate pollution), 2 ≤ PLI < 3 (heavy pollution), and PLI ≥ 3 (extremely heavy pollution, require immediate remediation).

#### 2.4.4. Index of Geo-Accumulation (Igeo)

To evaluate element accumulation in household dust, we calculated the index of geo-accumulation (Igeo), which assesses accumulation degree relative to geochemical background levels: [30,31] according to Equation (4):

(4) $$\mathrm{Igeo}\;=\;\log_{2}\frac{C_{n}}{1.5\;\times \;B_{n}}$$

where *C_n_* is the measured element concentration, *B_n_* is the geochemical background value from Taylor and McLennan, and factor 1.5 accounts for natural lithogenic variations [26,27]. Igeo values were classified as: Igeo ≤ 0 (unpolluted), 0 < Igeo ≤ 1 (unpolluted to moderately polluted), 1 < Igeo ≤ 2 (moderately polluted), 2 < Igeo ≤ 3 (moderately to heavily polluted), 3 < Igeo ≤ 4 (heavily polluted), 4 < Igeo ≤ 5 (heavily to extremely polluted), and Igeo > 5 (extremely polluted) [28,32].

### 2.5. Health Risk Assessment

Health risks from PTE exposure in household dust were estimated using the US Environmental Protection Agency (US EPA) model [5,23]. Three primary exposure routes were considered: ingestion, inhalation, and dermal contact. Both non-carcinogenic and carcinogenic risks were evaluated.

#### 2.5.1. Exposure Assessment

Exposure doses were calculated as average daily dose (ADD, mg kg^−1^ day^−1^) for each pathway using Equations (5)–(7) for noncarcinogenic risk and Equations (8)–(10) for carcinogenic risk [5,9,23].

The *ADD* for non-carcinogenic risk:

(5) $$ADD\;\mathrm{ingest}=\;C\;\times \;\frac{\mathrm{IngR}\;\times \;ExF\;\times \;ED}{BW\;\times \;AT}\;\times CF$$

(6) $$ADD\;\mathrm{inhal}=C\times \left[\frac{\;\mathrm{InhR}\times ExF\times ET\times ED}{PEF\times BW\times AT}\right]$$

(7) $$ADD\;\mathrm{dermal}=C\times \left[\frac{\;SA\times SL\times A\;BS\times ExF\times ED}{BW\times AT}\right]\times CF$$

The *ADD* for carcinogenic risk:

(8) $${ADD\;\mathrm{ingest}}_{\mathrm{carcinogenic}}=\;C\;\times \;\left[\frac{\;IR\;\times \;ExF\;}{AT}\right]\;\times CF$$

(9) $${ADD\;\mathrm{inhal}}_{\mathrm{carcinogenic}}=C\times \left[\frac{\;ExF\;\times ET\times ED}{PEF\;\times 24\times AT}\right]\times 1000$$

(10) $${ADD\;\mathrm{dermal}}_{\mathrm{carcinogenic}}=\;C\times \left[\frac{\;ABS\times ExF\times DFS}{AT}\right]\times CF$$

where *C* is the measured PTE concentration in household dust (mg kg^−1^); *IngR* and *InhR* are the ingestion (mg day^−1^) and inhalation rates (m^3^ day^−1^), respectively; *ExF* is the exposure frequency (days year^−1^); *ED* is the exposure duration; *BW* is the body weight (kg); *AT* is the average time (days); *PEF* is the particle emission factor (1.36 × 10^9^ m^3^ kg^−1^); *SL* is the skin adherence factor (mg cm^−2^); *SA* is the exposed skin area (cm^2^); *ABS* is the dermal absorption factor (unitless); *IR* is the ingestion rate for carcinogenic risk (Equation (S1)); and *DFS* is the adjusted soil dermal factor (mg year kg^−1^ day^−1^) [5,9,22,23].

Parameter values for health risk assessment were carefully selected to reflect local population characteristics and environmental conditions in UNT [9,22,23,33,34,35,36,37]. *BW* values were based on interview and previous study in UNT (adults: 60 kg, children: 15 kg). *IngR*, *ExF*, *PEF*, *SL*, and *SA* follow US EPA recommendations for residential scenarios [22]. *AT* and *DFS* were adopted from the international analysis studies [5,33]. *InhR*, *ED*, and *ABS* were derived from Asian population studies to represent regional physiological and behavioral characteristics [37]. These parameters collectively provide conservative, yet realistic exposure estimates for the Thai population in biomass-burning-impacted regions. All parameter values are listed in Tables S2 and S3.

#### 2.5.2. Non-Carcinogenic Risk Assessment

Non-carcinogenic risks were evaluated using the Hazard Quotient (HQ) and Hazard Index (HI) approaches [5]. Non-carcinogenic risks refer to adverse health effects from PTE exposure that do not involve cancer development. These effects may include neurological disorders, kidney damage, developmental problems, immune system dysfunction, and cardiovascular issues [38].

The calculated doses (*ADD*) of each element were compared to their corresponding reference dose (*RfD*) (mg kg^−1^ day^−1^) to yield a *HQ* for the three exposure pathways (Equation (11)), which were then summed as the total non-carcinogenic risk across all pathways using the hazard index (*HI*) (Equation (12)) [23]. For noncarcinogenic risk, *HQ* or *HI* < 1 indicates the absence of significant health effects. *HQ* or *HI* ≥ 1 indicates potential non-carcinogenic adverse health effects, with effects likely intensifying as *HQ* or *HI* increases [5,9,23,31].

(11) $$HQ=\frac{ADD\;}{RfD}$$

(12) $$HI=\sum {HQ}_{\mathrm{Ingest}}+{HQ}_{\mathrm{Inhal}}+{HQ}_{\mathrm{Dermal}}$$

#### 2.5.3. Carcinogenic Risk Assessment

The carcinogenic risk (CR) assesses lifetime carcinogenic effects by multiplying the dose by the relevant cancer slope factor (*SF*) [(mg kg^−1^ day^−1^)^−1^] (Equation (13)). The total cancer risk, or lifetime cancer risk (LCR), was evaluated by summing the cancer risk associated with each exposure pathway (Equation (14)) [5,9,23], as defined below:

(13) CR=ADD ×SF

(14) $$LCR=\sum {CR}_{Ingest}+{CR}_{Inhal}+{CR}_{Dermal}$$

Carcinogenic risk quantifies the probability that an individual will develop cancer over their lifetime due to exposure to carcinogenic PTEs. This assessment assumes that any exposure to carcinogenic PTEs carries some cancer risk, with no threshold below which risk is zero. For the carcinogenic risk, *CR* or *LCR* < 1 × 10^−6^ indicates the negligible risk (less than one additional cancer case per 1 million exposed individuals); 1 × 10^−6^ ≤ *CR* or *LCR* ≤ 1 × 10^−4^ indicates acceptable or tolerable risk; *CR* or *LCR* > 1 × 10^−4^ indicates unacceptable risk (more than one additional cancer cases per 10,000 exposed individuals), requiring Reference values for non-carcinogenic and carcinogenic risk assessment used in these models are shown in Table S3.

### 2.6. Statistical Analysis

Descriptive statistics (mean, median, standard deviation, and range) were calculated for all variables. Log-transformation was applied to all PTEs before normality assessment using the Shapiro–Wilk test. However, only As remained non-normally distributed even after log-transformation (*p* = 0.016). Differences between seasonal groups were evaluated using independent t-tests for normally distributed data and Mann–Whitney U tests for non-normally distributed data. Statistical significance was set at *p* < 0.05.

Principal Component Analysis (PCA) was performed to identify sources of PTEs in household dust. The Kaiser–Meyer–Olkin (KMO) test was used to assess sampling adequacy (target > 0.6), and Bartlett’s sphericity test confirmed the appropriateness of factor analysis (*p* < 0.05) [39]. Principal components with eigenvalues greater than 1.0 were retained following the Kaiser criterion. Varimax rotation with Kaiser normalization was applied to enhance interpretability of factor loadings.

## 3. Results and Discussion

### 3.1. Potentially Toxic Element Concentrations in Indoor Household Dust

**Figure 2:**
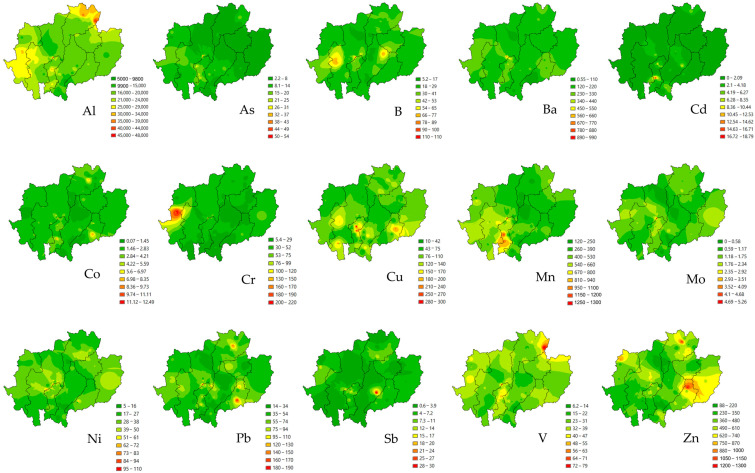
The distribution map of potentially toxic element concentration (mg kg^−1^) in upper northern Thailand.

Table 1 presents the concentrations of 15 potentially toxic elements (PTEs) in indoor household dust samples collected across eight provinces of upper northern Thailand and the distribution map of them were showed in Figure 2. Al exhibited the highest concentration (19,596 ± 7249 mg kg^−1^), followed by Mn (485 ± 229 mg kg^−1^), Zn (448 ± 281 mg kg^−1^), Ba (212 ± 109 mg kg^−1^), Cu (97.99 ± 72.37 mg kg^−1^), Pb (56.56 ± 43.78 mg kg^−1^), Cr (37.39 ± 34.23 mg kg^−1^), Ni (31.21 ± 20 mg kg^−1^), V (31.06 ± 12.37 mg kg^−1^), B (29.62 ± 21.02 mg kg^−1^), As (10.54 ± 7.13 mg kg^−1^), Sb (5.31 ± 5.22 mg kg^−1^), Co (2.41 ± 2.06 mg kg^−1^), Cd (1.93 ± 3.17 mg kg^−1^), and Mo (1.45 ± 0.83 mg kg^−1^). These concentrations were comparable to previous findings in Chiang Mai, Lamphun, and Lampang provinces, where indoor dust contamination patterns have been attributed to biomass burning emissions, vehicular traffic, industrial activities, mixed sources, and natural soil dust [20,21].

These findings align with previous studies that reported Al as the most abundant element in indoor dust globally (Table 2). However, elemental concentrations were lower than those in other countries, except for Mn and V [5,9,33,40,41]. The higher levels of Mn and V may result from outdoor contamination and soil resuspension, as these sources are highly enriched in Mn and V [5,41,42]. Moreover, most houses in UNT feature open-air designs that allow natural ventilation. Consequently, soil dust enriched in Mn and V can easily infiltrate indoor environments [19,20]. Additionally, agricultural contamination may be a potential source of Mn [43]. Most people in this region are engaged in agricultural work that can easily transfer agriculturally contaminated soil and chemicals into their bodies and homes [20].

Although PTE concentrations were within globally reported ranges, house dust composition varied considerably across locations, reflecting differences in local human activities and environmental conditions. Within Thailand, higher concentrations of Cd, Pb, and Zn have been reported in house dust near agricultural areas in Sukhothai Province [44] and roadside buildings in Phitsanulok Province [45]. Conversely, lower levels of Cr, Ni, and Pb were observed in houses near an electronic waste recycling area in Ubon Ratchathani [46], demonstrating the varied influence of anthropogenic activities on dust composition.

Nevertheless, As and Cr concentrations in house dust exceeded residential soil standard levels set by the Pollution Control Department of Thailand (PCD) [47]. Elevated As levels may be linked to agricultural lands and wooden houses that are abundant in UNT [5,48,49]. Elevated Cr levels may be associated with industrial activities in Lampang and Lamphun provinces [20,21]. However, the measured Cr concentration refers to total Cr, which is probably higher than the hexavalent form (Cr VI) alone recommended by PCD. Naturally, total Cr consists of only 0.8–4.5% Cr (VI) in general soils but can reach up to 90% of total Cr in leached soil areas [50,51,52].

**Table 2:** The comparisons of PTE analysis conducted in indoor household dust.

Country (*n*)	Potentially Toxic Element Concentrations (mg/kg) in Indoor Household Dust															Ref.
	Al	As	B	Ba	Cd	Co	Cr	Cu	Mn	Mo	Ni	Pb	Sb	V	Zn	
35 Countries (2265) ^a^		25.3			4.7		128	264	333		77.6	224			1470	[5]
35 Countries (2265) ^b^		13.3			0.8		86	176	257		39	94			1110	[5]
Canada (125) ^a^	16,000	13			11	5.4	92	1900	250	8.3	60	4500	36	15	14,000	[41]
Canada (125) ^b^	21,000	8.6			2.2	5.4	72	460	270	4.3	48	450	12	23	3100	[41]
China (3392) ^b^		15.6			2.7		86	136			40.7	161			603	[9]
Australia (224) ^a^		20.2					99.8	298	247		56.7	364			2437	[33]
Japan (100) ^b^	15,700			208	1.0	4.7	67.8	304	226	2.1	59.6	57.9	10.1	24.7	920	[40]
Thailand, Sukhothai (16) ^a^					9			2				226			1051	[44]
Thailand, Ubon Ratchathani																[46]
E–Waste (46) ^b^							1.5				1.2	6.9				
Non–E–Waste (46) ^b^							0.9				0.8	0.9				
Thailand, Phitsanulok																[45]
Main street (12) ^a^					21.3			89.9				128.7			519	
Secondary street (12) ^a^					24.7			67.7				62.3			322	
Thailand, Chiang Mai and Lamphun (100) ^b^		10.3			0.9		32.4	82.5	541		28.9	44.8			353	[20]
Thailand, Lampang ^b^		11.1			1.2		55.2	117.1	422		50.5	41.8		24.7	357	[21]
Thailand Soil Contamination		6			67		17.5 *	2920	1710		436.5	400				[47]
Thailand, UNT, Indoor ^a^	19,596	10.5	29.6	212	1.9	2.4	37.4	98.0	485	1.5	31.2	56.6	5.3	31.1	448	This study
Thailand, UNT, Indoor ^b^	18,826	8.8	26.5	194	1.0	1.9	31.4	74.4	429	1.3	26.8	42.6	3.3	29.8	374	

^a^ Mean, ^b^ Median, * Cr (VI).

### 3.2. Dust Pollution Assessment

#### 3.2.1. Enrichment Factor

Enrichment factor (EF) analysis revealed substantial anthropogenic contributions for several PTEs (Table 3). Sb demonstrated the highest EF value of 117.8, followed by Cd with an EF value of 88.2. EF values greater than 40 indicate extremely high anthropogenic input. As and Zn displayed significant enrichment with EF values of 30.2 and 28.4, respectively, indicating substantial anthropogenic contribution (20 ≤ EF < 40). Additionally, Cu, Pb, and B demonstrated moderate to significant enrichment with EF values of 17.5, 14.2, and 8.5, respectively, suggesting substantial anthropogenic input (5 ≤ EF < 20). Significant enrichment of these elements in indoor dust has been globally reported. Sb was primarily linked to flame retardants, worn consumer products, and other industrial applications [13,40,53]. As and Pb were reportedly related to agricultural lands and traffic emissions [48,54]. Meanwhile, Cd, Cu, Pb, and Zn were extensively associated with paint in houses [9,55]. On the other hand, minor enrichment (2 ≤ EF < 5) was observed in Mo, Mn, and Ni, indicating minimal anthropogenic influence with EF values of 4.2, 3.4, and 3.1, respectively. Mo and Ni were associated with stainless steel in household furniture [40,56]. Moderate to significant enrichment of these elements likely reflects contributions from other origins such as traffic emissions, paint deterioration, and construction materials [5]. In contrast, Cr, Ba, V, Al, and Co showed EF values below 2, suggesting predominantly natural sources. Al, Ba, and V were naturally associated with alkaline earth elements abundant in soils [41,57]. However, some elements originated from both anthropogenic and natural sources. As, Ni, Mn, and Zn were linked to household paint, traffic emissions, indoor smoking, as well as crustal soil dust [5,9,54,55,58,59]. Similar enrichment patterns in indoor house dust have been reported in human-activity-impacted areas such as industrial, agricultural, and urban zones [5,20,56], as well as in biomass-burning-impacted areas [21] where these elements are released during combustion processes and subsequently deposited in indoor environments.

#### 3.2.2. Contamination Factor and Pollution Load Index

Contamination factor analysis revealed considerable to very high contamination for several PTEs (Table 4). Sb showed the highest mean CF of 26.5, followed by Cd, As, and Zn at 19.7, 7.0, and 6.3, respectively, indicating very high contamination levels (CF ≥ 6). Cu and Pb indicated considerable contamination (3 ≤ CF < 6) with CF values of 3.9 and 3.3, respectively, while only B (CF = 2.0) fell into the moderate contamination range (1 ≤ CF < 3). The other PTEs, including Mo, Mn, Ni, Cr, Ba, V, Al, and Co, showed low contamination levels (CF < 1) with CF values of 1.0, 0.8, 0.7, 0.4, 0.4, 0.3, 0.2, and 0.1, respectively. The overall PLI was 0.7 ± 0.3, suggesting no pollution (PLI < 1) when considering all elements collectively.

Similarly, contamination factor analysis identified Sb, Cd, As, and Zn as priority pollutants, consistent with EF values indicating these PTEs originated predominantly from anthropogenic sources. Nevertheless, the overall PLI indicated no significant pollution when all PTEs were considered collectively. This apparent discrepancy reflects the fact that PLI represents a geometric mean of all contamination factors, and PTEs with low CF values can reduce the overall index despite high contamination by specific PTEs [32].

#### 3.2.3. Index of Geo-Accumulation

Igeo values indicated varying degrees of PTE accumulation in indoor household dust (Table 5). Sb exhibited the highest mean Igeo of 1.1, classified as moderately polluted (1 < Igeo ≤ 2). Cd, As, Zn, Cu, Pb, and B showed Igeo values of 0.9, 0.6, 0.6, 0.3, 0.3, and 0.1, respectively, indicating unpolluted to moderately polluted conditions (0 < Igeo ≤ 1). Most other elements, including Mo, Mn, Ni, Cr, Ba, V, Al, and Co, showed negative mean Igeo values, indicating unpolluted conditions (Igeo ≤ 0). Igeo values ranged only between unpolluted and moderately polluted categories. However, the wide range of Igeo values for Cd (0.16 to 2.14) and Sb (0.41 to 2.06) indicated substantial spatial variability, with some sampling locations experiencing moderate to heavy accumulation potentially related to proximity to emission sources or variations in household characteristics and activities [9,60,61].

Several PTEs, including As, B, Cd, Cu, Pb, Sb, and Zn, showed contamination in indoor house dust samples, indicating anthropogenic enrichment rather than natural sources. Nevertheless, Igeo values ranged only between unpolluted and moderately polluted levels, while the overall PLI suggested no pollution, with elemental concentrations remaining within background ranges. In contrast, other remaining PTEs showed low contamination levels, were unpolluted, and originated from natural sources. These findings indicate that contaminated PTEs in household dust were attributable to human activities, but levels had not reached thresholds that would constitute pollution [28,29].

#### 3.2.4. Contamination of PTEs in Household Dust During Air Pollution Seasons

Significant differences in PTE concentrations were observed between the haze and non-haze seasons for five elements (Table 6). Cr, Mo, Ni, Pb, and Zn concentrations were significantly higher during haze episodes compared to non-haze periods (*p* < 0.05). In contrast, no significant differences were found between the haze and non-haze seasons for the remaining PTEs (*p* > 0.05). These results are consistent with the impact of biomass burning on air quality in UNT where seasonal outdoor biomass burning produces severe elemental-bound particulate matter (PM) pollution annually [16,62,63]. During the haze season, PTEs can subsequently condense onto PM that infiltrates indoor environments through natural ventilation and deposits as settled dust [13,14,64].

**Figure 3:**
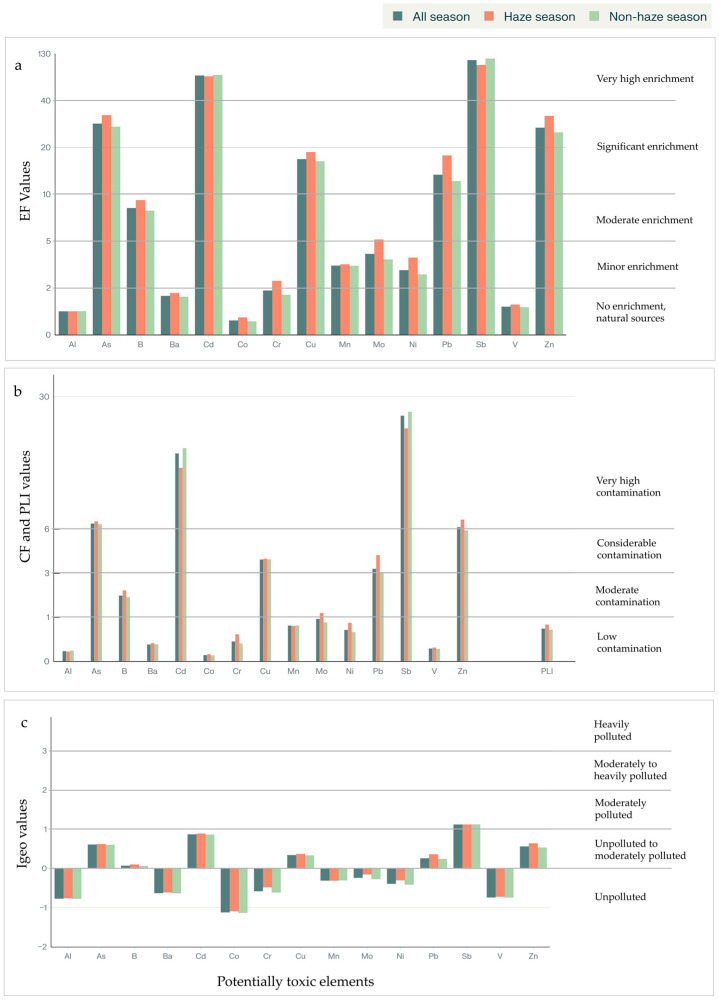
The contamination indices of 15 PTEs in household dust in UNT comparing the haze and non-haze seasons: (**a**) Enrichment Factor (EF); (**b**) Contamination Factor (CF) and Pollution Load Index (PLI); (**c**) Index of Geo-accumulation (Igeo).

Similarly to elemental concentrations, contamination factors (CF and PLI), enrichment factors (EF), and geo-accumulation indices (Igeo) showed significantly elevated values (*p* < 0.05) during the haze season for Cr, Mo, Ni, Pb, and Zn compared to the non-haze season (Figure 3 and Tables S4–S6). However, Al, As, B, Ba, Cd, Co, Cu, Mn, and Sb demonstrated no significant differences (*p* > 0.05) in EF and CF values between air pollution seasons. The overall pollution load index was also significantly higher during the haze season (0.8) than the non-haze season (0.7, *p* = 0.004).

The significant increase in EF, CF, PLI, and Igeo values during the haze season for these six metals indicates enhanced anthropogenic contamination during air pollution periods. Previous studies in northern Thailand have demonstrated that biomass burning is a major source of atmospheric PTEs, with elevated elemental concentrations in PM_2.5_ during air pollution season [16,65]. The absence of significant differences for the highly contaminated and enriched PTEs (As, Cd, and Sb) between seasons suggests these elements may originate from more consistent year-round sources rather than seasonal biomass burning [5,9].

### 3.3. Sources Analysis of Potentially Toxic Elements

To determine potential sources of PTEs in household dust in UNT, principal component analysis (PCA) was performed. The suitability of the dataset for PCA was confirmed by the Kaiser–Meyer–Olkin (KMO) measure of sampling adequacy and Bartlett’s test of sphericity. The overall KMO value was 0.749, indicating middling to good adequacy for factor analysis. Bartlett’s test yielded a significant result (χ^2^ = 805.590, degrees of freedom = 105, *p* < 0.001), confirming sufficient correlations among the 15 PTEs to justify PCA. Varimax rotation with Kaiser normalization was applied to facilitate interpretation. Using the criterion of eigenvalues greater than 1, five principal components (PCs) were extracted, collectively explaining 64.9% of the total variance in the dataset. The first component accounted for 17.2%, the second 16.8%, the third 10.9%, the fourth 10.4%, and the fifth 9.7% of the variance. Communalities after extraction indicated that the 15 PTEs were reasonably well represented by the retained components, with values ranging from 0.508 for Mn to 0.894 for Al, showing that each variable shared substantial variance with the component solution.

Table 7 presents the rotated component loadings of PCA for 15 PTEs in indoor household dust in UNT. The rotated loadings above 0.5 were considered significant for interpreting each component. PC1 was heavily loaded by Ni (0.759), Mo (0.685), Cu (0.655), B (0.633), and Cr (0.596), suggesting mixed sources including vehicle emissions, paint, and building materials in homes. Ni was associated with motor vehicles and decreases with distance from urban areas [5,55] while stainless steel furniture are sources of Mo and Cr [42,56]. Painting has been linked to Cu and Ni contamination in house dust [9,55,66]. Additionally, Ni, Cr, and Mo are related to worn consumer products such as stainless steel debris in homes [40]. The second component (PC2) had strong loadings for Al (0.894) and V (0.843), reflecting natural sources such as soil and crustal material [40,41,42]. PC3 was predominantly associated with Cd (0.663) and Co (0.718), indicating contributions from anthropogenic pollution including industrial activities, traffic emissions, and paint pigments [9,20,55,67]. PC4 loaded on As (0.738), Sb (0.686), and Mn (0.508), which may relate to agricultural inputs and worn consumer products [40,48]. Finally, PC5 featured significant loadings on Zn (0.823) and Pb (0.513), often linked to indoor smoking, wall painting, vehicle combustion, and biomass burning [21,55,58,68,69]. The five components reflect distinct potential sources or processes influencing PTE composition in house dust samples.

Concentration and contamination analyses revealed increased levels of Cr, Mo, Ni, Pb, V, and Zn during the haze season compared to the non-haze season, consistent with PM_2.5_ studies that found these PTEs were prominent during the haze seasons [16,65,70]. These six elements with increased concentrations were found dominantly in PC1 and PC5, indicating they are related to mixed sources. Interestingly, Pb and Zn, which have strong loadings in PC5, were also related to biomass burning in both PM_2.5_ and indoor dust [21,65,71]. Ni in PC1 was linked to wood and sugarcane combustion as well as mixed traffic-related sources [16,72]. These findings suggest that PC5 (Zn and Pb) and Ni in PC1 are associated with biomass burning in UNT

To identify potential seasonal variations in PTE sources and composition in house dust samples in UNT, the dataset was analyzed separately for the haze and non-haze seasons (Table 8). During the non-haze season, five PCs were extracted, collectively explaining 64.9% of the total variance. During the haze season, six PCs were retained, explaining 77.7% of the total variance, notably higher than the non-haze season. Communalities during the non-haze season ranged from 0.524 (Pb) to 0.812 (Al), indicating that most elements were well-represented by the extracted components. During the haze season, communalities ranged from 0.501 (Ba) to 0.921 (V), suggesting the component solution captured more variance, potentially reflecting more distinct pollution sources during this period.

PCA during the non-haze season indicated that PC1 (19.2% variance) was strongly loaded with Al (0.812) and V (0.791), suggesting crustal or soil-related sources [41,42]. PC2 (16.0% variance) was dominated by Ni (0.809), Cu (0.783), and Mo (0.618), indicating mixed sources of vehicle emissions, building, and decorative materials [5,9,40,55,56]. PC3 (12.0% variance) was highly loaded with As (0.811) and Mn (0.723), potentially related to agricultural sources [43,48]. PC4 (9.8% variance) was associated with Zn (0.749) and Sb (0.617), suggesting interior furniture and paint [13,53]. PC5 (7.9% variance) was characterized by Cd (0.772), indicating industrial activities, indoor smoking, and painting [9,20,55,59,73]. During the haze season, PC1 (18.8% variance) was strongly loaded with V (0.921), Mn (0.761), and Al (0.704), representing crustal elements and natural soils [5,41,42]. PC2 (16.7% variance) was dominated by Cu (0.769), Mo (0.738), Ni (0.636), and Cd (0.619), indicating intensified mixed sources of vehicle emissions, building, and decorative materials [5,9,40,55,56]. PC3 (13.1% variance) had high loadings for Pb (0.838) and Co (0.705), suggesting enhanced traffic combustion and biomass burning during the haze season [5,54,58,65]. PC4 (10.7% variance) was associated with Zn (0.850), reflecting biomass burning contamination [21,65,71]. PC5 (9.4% variance) was characterized by B (0.876) and Sb (0.557), potentially indicating indoor decorative materials such as glass fibers, metal jewelry, and plastic toys [13,40]. PC6 (9.0% variance) was uniquely loaded with As (0.908), possibly from enhanced wood and timber combustion and agricultural burning [5,21,48,74,75].

PC1 and PC2 in both the haze and non-haze seasons represented the same sources of natural soil and mixed sources, respectively, indicating baseline sources of these PTEs in house dust in UNT. However, the number of principal components increased from five during the non-haze season to six during the haze season, with total variance explained increasing from 64.9% to 77.7%. This suggests more diverse and distinct pollution sources during the haze season. The lower KMO value during the haze season (0.591 vs. 0.754) indicates greater complexity and potentially weaker inter-element correlations, reflecting the dynamic nature of pollution sources during air pollution scenarios [76,77]. The distinct separation of As and Zn components and the increased contribution of As and Zn during the haze season indicated additional sources associated with biomass burning in UNT [21,65,78].

### 3.4. Health Risk Assessment

#### 3.4.1. Non-Carcinogenic Risk Assessment

The non-carcinogenic risk assessment revealed distinct exposure patterns between adults and children across all three exposure pathways (Table 9). For adults, the total hazard index (HI) across all pathways was 3.26 × 10^−1^ (HQ_ing_ = 2.98 × 10^−1^, HQ_inh_ = 3.84 × 10^−3^, HQ_dermal_ = 2.41 × 10^−2^), indicating no significant health effects. Among individual PTEs, As contributed the highest HI of 7.26 × 10^−2^ for adults (HQ_ing_ = 5.61 × 10^−2^, HQ_inh_ = 1.35 × 10^−4^, HQ_dermal_ = 1.64 × 10^−2^), followed by Cr, Mn, Al, and Cd with an HI of 7.06 × 10^−2^, 3.59 × 10^−2^, 3.21 × 10^−2^, and 3.21 × 10^−2^, respectively. Nevertheless, children demonstrated substantially higher unacceptable non-carcinogenic risks than adults with a total HI of 2.51 (HQ_ing_ = 2.38, HQ_inh_ = 8.77 × 10^−3^, HQ_dermal_ = 1.15 × 10^−1^), exceeding the safe threshold of 1. This finding was consistent with previous studies on children’s vulnerability to indoor PTEs, which reflects their higher dust ingestion rates, lower body weights, and developing physiological systems [5,9,31,79]. Cr exhibited the highest HI for children at 5.51 × 10^−1^ (HQ_ing_ = 5.31 × 10^−1^, HQ_inh_ = 5.45 × 10^−4^, HQ_dermal_ = 1.89 × 10^−2^), followed by As, Mn, Cd, and Al with an HI of 5.27 × 10^−1^, 2.71 × 10^−1^, 2.53 × 10^−1^, and 2.52 × 10^−1^, respectively. However, other PTEs including Pb (HI = 2.10 × 10^−1^), Sb (HI = 1.70 × 10^−1^), Co (HI = 1.03 × 10^−1^), and V (HI = 7.89 × 10^−2^) also contributed to the total non-carcinogenic risk. The ingestion pathway consistently dominated non-carcinogenic risk exposure for both groups [5,9], accounting for 91.4% of total risk in adults and 95.1% in children, while inhalation contributed only 1.2% in adults and 0.3% in children, and dermal contact accounted for 7.4% in adults and 4.6% in children.

#### 3.4.2. Non-Carcinogenic Risk Assessment During Haze and Non-Haze Seasons

When comparing the haze and non-haze seasons, the total HI in adults was higher during the haze season than the non-haze season (Table 10). The total HI was 3.63 × 10^−1^ (HQ_ing_ = 3.32 × 10^−1^, HQ_inh_ = 4.11 × 10^−3^, HQ_dermal_ = 2.67 × 10^−2^) during the haze season, while it was 3.14 × 10^−1^ (HQ_ing_ = 2.87 × 10^−1^, HQ_inh_ = 3.77 × 10^−3^, HQ_dermal_ = 2.32 × 10^−2^) during the non-haze season. Nevertheless, the total HI remained below 1 across both seasons, indicating acceptable non-carcinogenic risk. Among these, Cr demonstrated the highest HI of 9.59 × 10^−2^ during the haze season (HQ_ing_ = 9.02 × 10^−2^, HQ_inh_ = 3.24 × 10^−4^, HQ_dermal_ = 5.40 × 10^−3^) compared to 6.21 × 10^−2^ during the non-haze season (HQ_ing_ = 5.84 × 10^−2^, HQ_inh_ = 2.10 × 10^−4^, HQ_dermal_ = 3.50 × 10^−3^), followed by As, Mn, and Pb with HI values increased from 7.08 × 10^−2^, 3.59 × 10^−2^, and 2.39 × 10^−2^ during the non-haze season to 7.77 × 10^−2^, 3.61 × 10^−2^, and 3.46 × 10^−2^ during the haze season, respectively.

In contrast, significantly elevated unacceptable non-carcinogenic risks in children were observed during the haze season (Table 11). The total hazard index for children increased from 2.41 during the non-haze season (HQ_ing_ = 2.29, HQ_inh_ = 8.60 × 10^−3^, HQ_dermal_ = 1.10 × 10^−1^) to 2.79 (HQ_ing_ = 2.66, HQ_inh_ = 9.38 × 10^−3^, HQ_dermal_ = 1.27 × 10^−1^) during the haze season. Cr demonstrated the highest HI of 7.48 × 10^−1^ during the haze season (HQ_ing_ = 7.21 × 10^−1^, HQ_inh_ = 7.40 × 10^−4^, HQ_dermal_ = 2.52 × 10^−2^) versus 4.85 × 10^−1^ during the non-haze season (HQ_ing_ = 4.67 × 10^−1^, HQ_inh_ = 4.80 × 10^−4^, HQ_dermal_ = 1.66 × 10^−2^). This was followed by As, Mn, Pb, and Al, which exhibited HI values of 5.64 × 10^−1^, 2.72 × 10^−1^, 2.74 × 10^−1^, and 2.55 × 10^−1^ during the haze season compared to 5.14 × 10^−1^, 2.71 × 10^−1^, 1.89 × 10^−1^, and 2.53 × 10^−1^ during the non-haze season.

Most PTEs showed higher HI values during the haze season, except for Cd and Sb, which demonstrated lower HI during the haze versus the non-haze seasons. This was due to higher concentrations of these PTEs during the non-haze season compared to the haze season, although no significant differences were found (Table 6). This seasonal variation reflects the enhanced deposition of biomass-burning-derived PTEs in house dust during air pollution periods related to PTE-bound PM_2.5_ [13,14,21,80]. Similar patterns have been observed in other biomass-burning-impacted regions, where children’s health risks from indoor dust exposure increase significantly during high-air-pollution scenarios [5,9,13,21].

#### 3.4.3. Carcinogenic Risk Assessment

To assess carcinogenic risk from PTE exposure in house dust, lifetime cancer risk assessment was conducted for six PTEs (As, Cd, Co, Cr, Ni, and Pb) across three exposure pathways (Table 12). Unacceptable carcinogenic risks were identified in both adults and children. For adults, Cr exhibited the highest total LCR of 4.22 × 10^−4^, particularly via inhalation (CR_ing_ = 6.79 × 10^−6^, CR_inh_ = 4.11 × 10^−4^, CR_dermal_ = 3.71 × 10^−6^), followed by As, Ni, Cd, Pb, and Co with LCR at 2.03 × 10^−5^, 5.60 × 10^−6^, 4.55 × 10^−6^, 6.05 × 10^−7^, and 5.68 × 10^−9^, respectively. The total lifetime cancer risk (total LCR) for adults was 4.53 × 10^−4^, which falls within the unacceptable risk range (CR > 1 × 10^−4^). For children, Cr exhibited the highest total LCR of 1.05 × 10^−4^ (CR_ing_ = 6.79 × 10^−6^, CR_inh_ = 9.50 × 10^−5^, CR_dermal_ = 3.71 × 10^−6^), representing unacceptable cancer risk, followed by As, Ni, Cd, Pb, and Co with LCR values of 2.03 × 10^−5^, 5.60 × 10^−6^, 1.80 × 10^−6^, 6.05 × 10^−7^, and 1.31 × 10^−9^, respectively. Only Cr LCR via overall pathways fell within the unacceptable risk range, leading to a total LCR for children of 1.34 × 10^−4^.

Carcinogenic risk assessment identified Cr as the primary driver of lifetime cancer risk for both adults and children, with both populations exceeding the acceptable risk threshold based on the US EPA guideline of 1 × 10^−4^. The dominance of Cr in carcinogenic risk, particularly through inhalation, has been reported in other indoor dust studies from regions with industrial activities, vehicular emissions, and biomass burning [5,9].

#### 3.4.4. Carcinogenic Risk Assessment During the Haze and Non-Haze Seasons

Carcinogenic risk comparison of PTEs between the haze and non-haze seasons in adults is shown in Table 13. Only Cr in adults exhibited unacceptable cancer risk, revealing the highest LCR values of 5.73 × 10^−4^ during the haze season (CR_ing_ = 9.22 × 10^−6^, CR_inh_ = 5.59 × 10^−4^, CR_dermal_ = 5.04 × 10^−6^) versus 3.71 × 10^−4^ during the non-haze season (CR_ing_ = 5.98 × 10^−6^, CR_inh_ = 3.62 × 10^−4^, CR_dermal_ = 3.27 × 10^−6^). The total LCR for adults increased from 4.02 × 10^−4^ during the non-haze season to 6.06 × 10^−4^ during the haze season, both remaining within the unacceptable risk range and predominantly driven by inhalation.

Interestingly, Cr in children exhibited the highest LCR of 9.28 × 10^−5^ during the non-haze season (CR_ing_ = 5.98 × 10^−6^, CR_inh_ = 8.36 × 10^−5^, CR_dermal_ = 3.27 × 10^−6^), which was within the acceptable risk range but elevated to unacceptable risk at 1.43 × 10^−4^ during the haze season (CR_ing_ = 9.22 × 10^−6^, CR_inh_ = 1.29 × 10^−4^, CR_dermal_ = 5.04 × 10^−6^) (Table 14). Only inhalation of Cr in household dust during the haze season indicated unacceptable cancer risk. The total LCR for children increased from 1.20 × 10^−4^ during the non-haze season to 1.74 × 10^−4^ during the haze season, with both values indicating unacceptable cancer risk. The elevated carcinogenic risk during the haze season was particularly pronounced for both adults and children. This seasonal elevation was driven primarily by increased Cr concentrations and exposure during the haze season.

Non-carcinogenic risk exceeding safe thresholds was found only in children, who exhibited risk levels 2.51 times the safe reference dose, predominantly via ingestion, with higher risks during the haze season. For carcinogenic risk, approximately 1.3 in 10,000 children and 4.5 in 10,000 adults may develop cancer from lifetime exposure to household dust PTEs. These risks were enhanced during the haze season, potentially reaching 1.7 in 10,000 children and 6.1 in 10,000 adults, particularly through inhalation.

The unacceptable health risks observed in this study were notably lower than those reported in some residential areas globally including Australia, New Caledonia, New Zealand, and some urban areas of China [5,9]. However, our values were higher than those reported in industrial regions of Thailand and Malaysia [20,21,81], coastal residential areas of Korea [82], and e-waste recycling sites in China [83]. This suggested that seasonal biomass burning in UNT creates substantial health risks comparable to some global residential areas, though with lower magnitude than certain high-exposure environments. It should be noted that health risk values depend on the number of elements included in calculations and element-specific reference dose values, which vary across studies. Different contamination sources also influence the proportion of elements considered in risk assessments. Furthermore, when compared with ambient PM_2.5_, household dust presented higher non-carcinogenic risk, whereas ambient PM_2.5_ exhibited substantially higher carcinogenic risk [84].

Within Southeast Asia, limited data exist for household dust health risks during biomass burning seasons. Our findings provide the first comprehensive seasonal risk assessment in a tropical biomass-burning-impacted region, filling critical knowledge gaps. The unacceptable risks identified for children (HI > 1, LCR > 10^−4^) across both seasons underscore the chronic nature of exposure, with seasonal biomass burning exacerbating already elevated baseline contamination from year-round anthropogenic sources.

The high unacceptable health risks for both adults and children, particularly during the haze season, highlight the public health significance of indoor dust contamination in UNT. These findings emphasize the need for integrated pollution control strategies addressing outdoor emission sources (particularly biomass burning) while reducing indoor contamination and associated health impacts [4,20,21,85,86].

## 4. Conclusions

This study provides comprehensive evidence of PTE contamination in indoor household dust across eight provinces in upper northern Thailand and reveals significant health risks, particularly during the haze season. Among the fifteen PTEs analyzed, Sb, Cd, As, and Zn exhibited very high contamination levels, while Cu and Pb showed considerable contamination. Enrichment factor analysis confirmed substantial anthropogenic contributions for these elements, demonstrating extremely high anthropogenic enrichment. Seasonal variations revealed significantly elevated concentrations of Cr, Mo, Ni, Pb, and Zn during the haze season compared to the non-haze season, accompanied by corresponding increases in contamination indices. Principal component analysis identified five distinct sources including mixed sources (17.2%), natural soils (16.8%), traffic combustion, industrial activities and wall paint (10.9%), agricultural lands and house’s decoration (10.4%), and smoking, paint and biomass burning (9.7%). Seasonal variations identified five distinct sources during the non-haze season and six sources during the haze season, explaining 64.9% and 77.7% of total variance, respectively. The emergence of additional components during the haze season confirmed biomass burning as a significant seasonal contributor to indoor dust contamination in this region.

Health risk assessment demonstrated unacceptable non-carcinogenic risks exclusively for children with total HI of 2.51, and increasing to 2.79 during the haze season, both exceeding the safe threshold of 1.0. Ingestion was identified as the primary exposure pathway for non-carcinogenic risks. Carcinogenic risk assessment revealed that Cr posed unacceptable lifetime cancer risks for both adults (LCR = 4.22 × 10^−4^) and children (LCR = 1.05 × 10^−4^), predominantly through inhalation. Total LCR increased substantially during the haze season, rising from 4.02 × 10^−4^ to 6.06 × 10^−4^ in adults and from 1.20 × 10^−4^ to 1.74 × 10^−4^ in children. This study demonstrates an increase in health risks from PTE exposure during the height of seasonal air pollution episodes in UNT, even though it occurs through indoor household dust. These findings underscore the critical need for public health interventions in UNT, particularly during annual haze episodes when children face substantially elevated health risks from indoor dust ingestion and all populations experience heightened cancer risks via Cr inhalation. Mitigation strategies should prioritize reducing indoor dust accumulation through enhanced ventilation filtration systems, regular cleaning protocols, and source controls targeting biomass burning emissions.

This research contributes novel insights by presenting the first comprehensive multi-provincial and seasonal assessment of indoor dust PTEs in a biomass-burning-impacted tropical region, quantitatively demonstrating biomass burning’s contribution through cross-seasonal comparison and source apportionment, and revealing that seasonal pollution episodes significantly elevate annual health risks beyond baseline levels. Future research should prioritize longitudinal monitoring, intervention studies on air purifiers and cleaning protocols during haze, biomonitoring, and regional modeling to support evidence-based policies protecting vulnerable populations in biomass-burning regions.

## Figures and Tables

**Table 1:** The concentrations of PTEs (mg kg^−1^) in indoor household dust.

PTEs	Concentration (mg kg^−1^)						
	*n*	Mean	SD	Median	IQR	Min.	Max.
Al	210	19,596	7249	18,826	6841	2161	75,634
As	207	10.54	7.13	8.75	6.18	2.23	62.13
B	209	29.62	21.02	26.47	9.78	8.70	184.20
Ba	207	211.64	109.26	194.04	71.47	20.71	996.45
Cd	194	1.93	3.17	0.95	1.25	0.21	20.24
Co	206	2.41	2.06	1.94	1.63	0.42	17.05
Cr	210	37.39	34.23	31.41	16.69	3.02	465.47
Cu	205	97.99	72.37	74.41	51.69	13.72	438.75
Mn	210	485.25	228.99	428.88	238.27	30.07	2041
Mo	201	1.45	0.83	1.27	0.75	0.60	8.55
Ni	210	31.21	20.00	26.83	14.19	2.24	146.24
Pb	208	56.56	43.78	42.62	31.23	4.21	302.61
Sb	203	5.31	5.22	3.32	3.10	0.77	34.79
V	210	31.06	12.37	29.77	12.86	2.91	120.87
Zn	207	447.79	280.60	374.17	225.44	56.97	1779

**Table 3:** The Enrichment Factor (EF) of PTEs in indoor household dust.

PTEs	Enrichment Factor (EF)						
	*n*	Mean	SD	Median	IQR	Min.	Max.
Al	210	1.00	-	1.00	-	1.00	1.00
As	207	30.17	27.39	25.68	15.04	9.26	332.22
B	209	8.53	6.17	7.40	3.88	2.05	72.82
Ba	207	1.66	0.90	1.51	0.60	0.51	9.22
Cd	194	88.23	148.14	43.11	61.86	4.65	1062.08
Co	206	0.61	0.55	0.47	0.35	0.07	4.82
Cr	210	1.89	1.29	1.57	0.82	0.72	14.22
Cu	205	17.50	13.77	13.49	11.59	4.74	97.73
Mn	210	3.43	1.37	3.19	1.76	1.32	9.18
Mo	201	4.18	2.33	3.56	2.26	1.11	15.46
Ni	210	3.14	2.17	2.63	1.83	0.75	15.64
Pb	208	14.17	10.65	10.61	7.98	3.11	74.63
Sb	203	117.80	127.90	74.81	69.50	21.96	995.01
V	210	1.20	0.24	1.20	0.32	0.68	2.26
Zn	207	28.44	20.06	22.81	18.88	6.69	144.07

**Table 4:** The contamination factor and pollution load index (CF and PLI) of PTEs in indoor household dust.

PTEs	Contamination Factor (CF) and Pollution Load Index (PLI)						
	*n*	Mean	SD	Median	IQR	Min.	Max.
Al	209	0.24	0.09	0.23	0.08	0.03	0.94
As	206	6.98	4.73	5.82	4.11	1.49	41.42
B	208	1.98	1.40	1.77	0.65	0.58	12.28
Ba	206	0.38	0.20	0.35	0.13	0.04	1.81
Cd	193	19.70	32.45	9.69	12.33	2.16	206.54
Co	205	0.14	0.12	0.11	0.10	0.02	1.00
Cr	209	0.45	0.41	0.38	0.20	0.04	5.61
Cu	204	3.92	2.90	2.97	2.08	0.55	17.55
Mn	209	0.81	0.38	0.71	0.40	0.05	3.40
Mo	200	0.96	0.56	0.84	0.49	0.40	5.70
Ni	209	0.71	0.46	0.61	0.33	0.05	3.32
Pb	207	3.29	2.53	2.50	1.79	0.25	17.80
Sb	203	26.55	26.10	16.61	15.50	3.84	173.96
V	209	0.29	0.12	0.28	0.12	0.03	1.13
Zn	206	6.32	3.96	5.29	3.18	0.80	25.05
PLI	209	0.74	0.28	0.71	0.26	0.03	3.41

**Table 5:** The index of geo-accumulation (Igeo) of PTEs in indoor household dust.

PTEs	Index of Geo-Accumulation (Igeo)						
	*n*	Mean	SD	Median	IQR	Min.	Max.
Al	210	−0.81	0.15	−0.80	0.17	−1.75	0.00
As	207	0.61	0.22	0.59	0.30	0.00	1.44
B	209	0.07	0.18	0.07	0.17	−0.41	0.91
Ba	207	−0.63	0.17	−0.63	0.16	−1.60	0.08
Cd	194	0.87	0.41	0.81	0.51	0.16	2.14
Co	206	−1.12	0.28	−1.12	0.36	−1.78	−0.17
Cr	210	−0.58	0.20	−0.60	0.22	−1.61	0.57
Cu	205	0.34	0.25	0.30	0.28	−0.44	1.07
Mn	209	−0.31	0.19	−0.32	0.23	−1.48	0.36
Mo	201	−0.24	0.19	−0.25	0.25	−0.57	0.58
Ni	210	−0.39	0.23	−0.39	0.23	−1.47	0.35
Pb	208	0.26	0.26	0.22	0.30	−0.78	1.07
Sb	203	1.12	0.30	1.04	0.34	0.41	2.06
V	210	−0.74	0.16	−0.73	0.19	−1.74	−0.12
Zn	207	0.56	0.23	0.55	0.27	−0.27	1.22

**Table 6:** Concentrations of PTEs (mg kg^−1^) in indoor household dust comparing the haze and non-haze seasons.

PTEs	Concentrations (mg kg^−1^)										*p*-Value
	Haze Season					Non-Haze Season					
	*n*	Mean	SD	Median	IQR	*n*	Mean	SD	Median	IQR	
Al	54	19,795	10,391	18,575	6904	154	19,651.14	5687.81	18,887.20	6696.24	0.281
As	54	11.27	9.39	8.82	6.24	152	10.26	6.17	8.59	6.33	0.788
B	54	32.85	25.67	27.26	11.33	154	28.51	19.17	26.10	9.83	0.178
Ba	54	224.55	151.32	194.43	65.92	151	207.91	89.25	193.99	73.14	0.623
Cd	52	1.68	2.32	1.11	1.43	141	2.02	3.45	0.85	1.01	0.617
Co	53	2.77	2.78	2.10	2.22	152	2.29	1.74	1.92	1.52	0.328
Cr	54	50.77	61.25	41.15	17.36	154	32.90	14.47	29.53	12.88	0.000
Cu	52	99.36	53.95	80.37	54.09	151	98.34	77.95	72.10	51.97	0.358
Mn	54	488.31	271.49	431.93	214.46	154	485.82	210.87	424.88	239.00	0.891
Mo	52	1.79	1.15	1.52	0.81	148	1.32	0.65	1.16	0.63	0.000
Ni	54	38.17	25.65	29.75	21.83	154	28.90	17.03	24.88	13.56	0.001
Pb	54	73.85	60.63	48.68	52.53	152	50.90	34.30	41.91	28.41	0.007
Sb	51	4.85	3.25	3.32	3.84	151	5.45	5.75	3.32	2.54	0.925
V	54	33.24	16.08	32.60	16.68	154	30.49	10.59	29.49	11.51	0.407
Zn	54	648.71	872.15	418.27	348.60	152	449.47	445.50	357.63	225.60	0.004

**Table 7:** The rotated component loadings of principal component analysis (PCA) for 15 PTEs in indoor household dust samples in UNT.

PTEs	Component				
	1	2	3	4	5
Al	−0.040	0.894	−0.076	0.024	0.032
As	0.043	0.252	0.113	0.738	−0.163
B	0.633	0.173	−0.254	0.051	−0.207
Ba	0.106	0.461	0.373	0.363	0.105
Cd	0.027	−0.164	0.663	0.188	0.124
Co	0.330	0.236	0.718	−0.094	−0.263
Cr	0.596	0.497	0.214	0.057	0.226
Cu	0.655	−0.080	0.122	0.147	0.229
Mn	0.148	0.464	0.293	0.508	−0.333
Mo	0.685	0.179	0.148	0.175	0.195
Ni	0.759	0.013	0.267	0.056	0.096
Pb	0.181	0.354	0.460	0.019	0.513
Sb	0.263	−0.047	−0.040	0.686	0.286
V	0.224	0.843	−0.004	0.205	−0.032
Zn	0.211	−0.009	−0.030	−0.002	0.823
Eigenvalues	2.574	2.527	1.630	1.555	1.450
% Of variance	17.16	16.84	10.87	10.36	9.67
Cumulative %	17.16	34.00	44.87	55.23	64.90
Estimate sources	Mixed sources of vehicle emission, decorative materials, and biomass burning	Natural soils	Traffic combustion, industrial activities and wall paint	Agricultural lands and house’s decoration	Smoking, paint and biomass burning

**Table 8:** The rotated component loadings of principal component analysis for 15 PTEs in indoor household dust in UNT across the haze and non-haze seasons.

PTEs	Component										
	Haze Season						Non-Haze Season				
	1	2	3	4	5	6	1	2	3	4	5
Al	0.704	−0.292	0.109	0.448	0.190	0.072	0.812	−0.020	0.116	−0.194	−0.117
As	0.199	0.044	−0.013	−0.027	−0.076	0.908	0.168	0.145	0.811	−0.046	0.070
B	0.164	0.213	−0.080	−0.064	0.876	−0.149	0.248	0.289	0.124	0.112	−0.560
Ba	0.505	0.237	0.501	−0.065	−0.032	0.396	0.585	0.039	0.269	0.372	0.084
Cd	−0.164	0.619	0.151	−0.403	0.151	0.084	0.086	0.125	0.126	0.118	0.772
Co	0.095	0.289	0.705	−0.425	−0.067	−0.252	0.498	0.363	0.193	−0.254	0.262
Cr	0.596	0.242	0.293	0.302	0.233	0.271	0.597	0.579	0.032	0.123	0.007
Cu	−0.050	0.769	0.184	0.176	0.078	0.165	−0.039	0.783	0.069	0.112	−0.010
Mn	0.761	0.282	0.022	−0.271	−0.271	0.064	0.419	0.141	0.723	−0.070	0.025
Mo	0.423	0.738	−0.061	0.209	0.054	−0.052	0.281	0.618	0.151	0.294	−0.071
Ni	0.050	0.636	0.345	0.040	0.278	−0.046	0.115	0.809	0.114	0.026	0.033
Pb	0.082	0.095	0.838	0.291	0.026	0.083	0.524	0.228	−0.032	0.368	0.338
Sb	0.006	0.324	0.466	0.049	0.557	0.379	−0.063	0.103	0.516	0.617	−0.149
V	0.921	−0.092	0.026	0.019	0.208	0.092	0.791	0.159	0.271	0.043	−0.150
Zn	0.023	0.220	0.097	0.850	−0.048	−0.041	−0.005	0.240	−0.288	0.749	0.128
Eigenvalues	2.827	2.506	1.962	1.604	1.408	1.350	2.875	2.405	1.800	1.468	1.186
% Of variance	18.85	16.70	13.08	10.69	9.39	9.00	19.17	16.03	12.00	9.79	7.91
Cumulative %	18.85	35.55	48.63	59.32	68.71	77.71	19.17	35.20	47.20	56.99	64.90

**Table 9:** Non-carcinogenic risk or hazard quotient (HQ) of 15 PTEs in indoor household dust in UNT.

PTEs	Non-Carcinogenic Risk							
	Adult				Children			
	HQ_ing_	HQ_inh_	HQ_dermal_	HI	HQ_ing_	HQ_inh_	HQ_dermal_	HI
Al	3.13 × 10^−2^	7.51 × 10^−4^		3.21 × 10^−2^	2.51 × 10^−1^	1.71 × 10^−3^		2.52 × 10^−1^
As	5.61 × 10^−2^	1.35 × 10^−4^	1.64 × 10^−2^	7.26 × 10^−2^	4.49 × 10^−1^	3.07 × 10^−4^	7.80 × 10^−2^	5.27 × 10^−1^
B	2.37 × 10^−4^	2.84 × 10^−7^		2.37 × 10^−4^	1.89 × 10^−3^	6.48 × 10^−7^		1.89 × 10^−3^
Ba	1.69 × 10^−3^	8.11 × 10^−5^		1.77 × 10^−3^	1.35 × 10^−2^	1.85 × 10^−4^		1.37 × 10^−2^
Cd	3.09 × 10^−2^	3.70 × 10^−5^	1.23 × 10^−3^	3.21 × 10^−2^	2.47 × 10^−1^	8.45 × 10^−5^	5.86 × 10^−3^	2.53 × 10^−1^
Co	1.28 × 10^−2^	7.69 × 10^−5^	9.60 × 10^−7^	1.29 × 10^−2^	1.03 × 10^−1^	1.76 × 10^−4^	4.57 × 10^−6^	1.03 × 10^−1^
Cr	6.64 × 10^−2^	2.39 × 10^−4^	3.97 × 10^−3^	7.06 × 10^−2^	5.31 × 10^−1^	5.45 × 10^−4^	1.89 × 10^−2^	5.51 × 10^−1^
Cu	3.92 × 10^−3^	4.67 × 10^−7^	5.21 × 10^−5^	3.97 × 10^−3^	3.13 × 10^−2^	1.07 × 10^−6^	2.48 × 10^−4^	3.16 × 10^−2^
Mn	3.23 × 10^−2^	1.86 × 10^−3^	1.68 × 10^−3^	3.59 × 10^−2^	2.59 × 10^−1^	4.24 × 10^−3^	8.00 × 10^−3^	2.71 × 10^−1^
Mo	4.63 × 10^−4^	1.39 × 10^−7^		4.63 × 10^−4^	3.70 × 10^−3^	3.17 × 10^−7^		3.70 × 10^−3^
Ni	2.49 × 10^−3^	5.98 × 10^−4^	3.69 × 10^−5^	3.13 × 10^−3^	1.99 × 10^−2^	1.36 × 10^−3^	1.75 × 10^−4^	2.15 × 10^−2^
Pb	2.58 × 10^−2^	3.08 × 10^−6^	6.87 × 10^−4^	2.65 × 10^−2^	2.07 × 10^−1^	7.03 × 10^−6^	3.27 × 10^−3^	2.10 × 10^−1^
Sb	2.12 × 10^−2^	3.39 × 10^−6^		2.12 × 10^−2^	1.70 × 10^−1^	7.74 × 10^−6^		1.70 × 10^−1^
V	9.85 × 10^−3^	5.95 × 10^−5^		9.91 × 10^−3^	7.88 × 10^−2^	1.36 × 10^−4^		7.89 × 10^−2^
Zn	2.39 × 10^−3^	2.86 × 10^−7^	4.76 × 10^−5^	2.43 × 10^−3^	1.91 × 10^−2^	6.53 × 10^−7^	2.26 × 10^−4^	1.93 × 10^−2^
Total	2.98 × 10^−1^	3.84 × 10^−3^	2.41 × 10^−2^	3.26 × 10^−1^	2.38	8.77 × 10^−3^	1.15 × 10^−1^	2.51

**Table 10:** Non-carcinogenic risk or hazard quotient (HQ) of 15 PTEs in indoor household dust in UNT in adults.

PTEs	Non-Carcinogenic Risk (Adults)							
	Haze Season				Non-Haze Season			
	HQ_ing_	HQ_inh_	HQ_dermal_	HI	HQ_ing_	HQ_inh_	HQ_dermal_	HI
Al	3.16 × 10^−2^	7.59 × 10^−4^		3.24 × 10^−2^	3.14 × 10^−2^	7.53 × 10^−4^		3.22 × 10^−2^
As	6.00 × 10^−2^	1.44 × 10^−4^	1.75 × 10^−2^	7.77 × 10^−2^	5.47 × 10^−2^	1.31 × 10^−4^	1.60 × 10^−2^	7.08 × 10^−2^
B	2.63 × 10^−4^	3.15 × 10^−7^		2.63 × 10^−4^	2.28 × 10^−4^	2.73 × 10^−7^		2.28 × 10^−4^
Ba	1.79 × 10^−3^	8.61 × 10^−5^		1.88 × 10^−3^	1.66 × 10^−3^	7.97 × 10^−5^		1.74 × 10^−3^
Cd	2.69 × 10^−2^	3.23 × 10^−5^	1.07 × 10^−3^	2.80 × 10^−2^	3.23 × 10^−2^	3.88 × 10^−5^	1.29 × 10^−3^	3.37 × 10^−2^
Co	1.48 × 10^−2^	8.85 × 10^−5^	1.10 × 10^−6^	1.48 × 10^−2^	1.22 × 10^−2^	7.30 × 10^−5^	9.11 × 10^−7^	1.23 × 10^−2^
Cr	9.02 × 10^−2^	3.24 × 10^−4^	5.40 × 10^−3^	9.59 × 10^−2^	5.84 × 10^−2^	2.10 × 10^−4^	3.50 × 10^−3^	6.21 × 10^−2^
Cu	3.97 × 10^−3^	4.74 × 10^−7^	5.28 × 10^−5^	4.02 × 10^−3^	3.93 × 10^−3^	4.69 × 10^−7^	5.23 × 10^−5^	3.98 × 10^−3^
Mn	3.25 × 10^−2^	1.87 × 10^−3^	1.69 × 10^−3^	3.61 × 10^−2^	3.24 × 10^−2^	1.86 × 10^−3^	1.68 × 10^−3^	3.59 × 10^−2^
Mo	5.74 × 10^−4^	1.72 × 10^−7^		5.74 × 10^−4^	4.22 × 10^−4^	1.27 × 10^−7^		4.22 × 10^−4^
Ni	3.05 × 10^−3^	7.31 × 10^−4^	4.51 × 10^−5^	3.83 × 10^−3^	2.31 × 10^−3^	5.54 × 10^−4^	3.41 × 10^−5^	2.90 × 10^−3^
Pb	3.37 × 10^−2^	4.02 × 10^−6^	8.97 × 10^−4^	3.46 × 10^−2^	2.32 × 10^−2^	2.77 × 10^−6^	6.18 × 10^−4^	2.39 × 10^−2^
Sb	1.94 × 10^−2^	3.10 × 10^−6^		1.94 × 10^−2^	2.18 × 10^−2^	3.48 × 10^−6^		2.18 × 10^−2^
V	1.05 × 10^−2^	6.37 × 10^−5^		1.06 × 10^−2^	9.67 × 10^−3^	5.84 × 10^−5^		9.73 × 10^−3^
Zn	2.88 × 10^−3^	3.45 × 10^−7^	5.74 × 10^−5^	2.93 × 10^−3^	2.23 × 10^−3^	2.67 × 10^−7^	4.45 × 10^−5^	2.27 × 10^−3^
Total	3.32 × 10^−1^	4.11 × 10^−3^	2.67 × 10^−2^	3.63 × 10^−1^	2.87 × 10^−1^	3.77 × 10^−3^	2.32 × 10^−2^	3.14 × 10^−1^

**Table 11:** Non-carcinogenic risk or hazard quotient (HQ) of 15 PTEs in indoor household dust in UNT in children.

PTEs	Non-Carcinogenic Risk (Children)							
	Haze Season				Non-Haze Season			
	HQ_ing_	HQ_inh_	HQ_dermal_	HI	HQ_ing_	HQ_inh_	HQ_dermal_	HI
Al	2.53 × 10^−1^	1.73 × 10^−3^		2.55 × 10^−1^	2.51 × 10^−1^	1.72 × 10^−3^		2.53 × 10^−1^
As	4.80 × 10^−1^	3.29 × 10^−4^	8.34 × 10^−2^	5.64 × 10^−1^	4.37 × 10^−1^	2.99 × 10^−4^	7.59 × 10^−2^	5.14 × 10^−1^
B	2.10 × 10^−3^	7.18 × 10^−7^		2.10 × 10^−3^	1.82 × 10^−3^	6.23 × 10^−7^		1.82 × 10^−3^
Ba	1.44 × 10^−2^	1.96 × 10^−4^		1.46 × 10^−2^	1.33 × 10^−2^	1.82 × 10^−4^		1.35 × 10^−2^
Cd	2.15 × 10^−1^	7.36 × 10^−5^	5.11 × 10^−3^	2.20 × 10^−1^	2.59 × 10^−1^	8.85 × 10^−5^	6.14 × 10^−3^	2.65 × 10^−1^
Co	1.18 × 10^−1^	2.02 × 10^−4^	5.25 × 10^−6^	1.18 × 10^−1^	9.75 × 10^−2^	1.67 × 10^−4^	4.34 × 10^−6^	9.76 × 10^−2^
Cr	7.21 × 10^−1^	7.40 × 10^−4^	2.57 × 10^−2^	7.48 × 10^−1^	4.67 × 10^−1^	4.80 × 10^−4^	1.66 × 10^−2^	4.85 × 10^−1^
Cu	3.18 × 10^−2^	1.08 × 10^−6^	2.51 × 10^−4^	3.20 × 10^−2^	3.14 × 10^−2^	1.07 × 10^−6^	2.49 × 10^−4^	3.17 × 10^−2^
Mn	2.60 × 10^−1^	4.27 × 10^−3^	8.05 × 10^−3^	2.72 × 10^−1^	2.59 × 10^−1^	4.25 × 10^−3^	8.01 × 10^−3^	2.71 × 10^−1^
Mo	4.59 × 10^−3^	3.92 × 10^−7^		4.59 × 10^−3^	3.38 × 10^−3^	2.89 × 10^−7^		3.38 × 10^−3^
Ni	2.44 × 10^−2^	1.67 × 10^−3^	2.14 × 10^−4^	2.63 × 10^−2^	1.85 × 10^−2^	1.26 × 10^−3^	1.62 × 10^−4^	1.99 × 10^−2^
Pb	2.70 × 10^−1^	9.18 × 10^−6^	4.27 × 10^−3^	2.74 × 10^−1^	1.86 × 10^−1^	6.32 × 10^−6^	2.94 × 10^−3^	1.89 × 10^−1^
Sb	1.55 × 10^−1^	7.06 × 10^−6^		1.55 × 10^−1^	1.74 × 10^−1^	7.94 × 10^−6^		1.74 × 10^−1^
V	8.43 × 10^−2^	1.45 × 10^−4^		8.45 × 10^−2^	7.73 × 10^−2^	1.33 × 10^−4^		7.75 × 10^−2^
Zn	2.30 × 10^−2^	7.87 × 10^−7^	2.73 × 10^−4^	2.33 × 10^−2^	1.78 × 10^−2^	6.10 × 10^−7^	2.12 × 10^−4^	1.80 × 10^−2^
Total	2.66	9.38 × 10^−3^	1.27 × 10^−1^	2.79	2.29	8.60 × 10^−3^	1.10 × 10^−1^	2.41

**Table 12:** Carcinogenic risk (CR) and lifetime cancer risk (LCR) of 6 PTEs in indoor household dust in UNT.

PTEs	Carcinogenic Risk							
	Adult				Child			
	CR_ing_	CR_inh_	CR_dermal_	LCR	CR_ing_	CR_nh_	CR_dermal_	LCR
As	1.79 × 10^−5^	1.19 × 10^−8^	2.35 × 10^−6^	2.03 × 10^−5^	1.79 × 10^−5^	2.74 × 10^−9^	2.35 × 10^−6^	2.03 × 10^−5^
Cd	8.33 × 10^−7^	3.57 × 10^−6^	1.46 × 10^−7^	4.55 × 10^−6^	8.33 × 10^−7^	8.23 × 10^−7^	1.46 × 10^−7^	1.80 × 10^−6^
Co		5.68 × 10^−9^		5.68 × 10^−9^		1.31 × 10^−9^		1.31 × 10^−9^
Cr	6.79 × 10^−6^	4.11 × 10^−4^	3.71 × 10^−6^	4.22 × 10^−4^	6.79 × 10^−6^	9.50 × 10^−5^	3.71 × 10^−6^	1.05 × 10^−4^
Ni	5.46 × 10^−6^	2.13 × 10^−9^	1.41 × 10^−7^	5.60 × 10^−6^	5.46 × 10^−6^	4.91 × 10^−10^	1.41 × 10^−7^	5.60 × 10^−6^
Pb	5.46 × 10^−7^	1.78 × 10^−10^	5.90 × 10^−8^	6.05 × 10^−7^	5.46 × 10^−7^	4.10 × 10^−11^	5.90 × 10^−8^	6.05 × 10^−7^
Total	3.16 × 10^−5^	4.15 × 10^−4^	6.41 × 10^−6^	4.53 × 10^−4^	3.16 × 10^−5^	9.58 × 10^−5^	6.41 × 10^−6^	1.34 × 10^−4^

**Table 13:** Carcinogenic risk (CR) and lifetime cancer risk (LCR) of 6 PTEs in indoor household dust in UNT in adults.

PTEs	Carcinogenic Risk (Adults)							
	Haze Season				Non-Haze Season			
	CR_ing_	CR_inh_	CR_dermal_	LCR	CR_ing_	CR_nh_	CR_dermal_	LCR
As	1.92 × 10^−5^	1.27 × 10^−8^	2.52 × 10^−6^	2.17 × 10^−5^	1.75 × 10^−5^	1.16 × 10^−8^	2.29 × 10^−6^	1.98 × 10^−5^
Cd	7.26 × 10^−7^	3.11 × 10^−6^	1.27 × 10^−7^	3.96 × 10^−6^	8.73 × 10^−7^	3.74 × 10^−6^	1.53 × 10^−7^	4.76 × 10^−6^
Co		6.53 × 10^−9^		6.53 × 10^−9^		5.39 × 10^−9^		5.39 × 10^−9^
Cr	9.22 × 10^−6^	5.59 × 10^−4^	5.04 × 10^−6^	5.73 × 10^−4^	5.98 × 10^−6^	3.62 × 10^−4^	3.27 × 10^−6^	3.71 × 10^−4^
Ni	6.67 × 10^−6^	2.60 × 10^−9^	1.72 × 10^−7^	6.85 × 10^−6^	5.05 × 10^−6^	1.97 × 10^−9^	1.31 × 10^−7^	5.18 × 10^−6^
Pb	7.13 × 10^−7^	2.32 × 10^−10^	7.70 × 10^−8^	7.90 × 10^−7^	4.91 × 10^−7^	1.60 × 10^−10^	5.31 × 10^−8^	5.44 × 10^−7^
Total	3.65 × 10^−5^	5.62 × 10^−4^	7.94 × 10^−6^	6.06 × 10^−4^	2.99 × 10^−5^	3.66 × 10^−4^	5.90 × 10^−6^	4.02 × 10^−4^

**Table 14:** Carcinogenic risk (CR) and lifetime cancer risk (LCR) of 6 PTEs in indoor household dust in UNT in children.

PTEs	Carcinogenic Risk (Children)							
	Haze Season				Non-Haze Season			
	CR_ing_	CR_inh_	CR_dermal_	LCR	CR_ing_	CR_nh_	CR_dermal_	LCR
As	1.92 × 10^−5^	2.93 × 10^−9^	2.52 × 10^−6^	2.17 × 10^−5^	1.75 × 10^−5^	2.67 × 10^−9^	2.29 × 10^−6^	1.98 × 10^−5^
Cd	7.26 × 10^−7^	7.17 × 10^−7^	1.27 × 10^−7^	1.57 × 10^−6^	8.73 × 10^−7^	8.63 × 10^−7^	1.53 × 10^−7^	1.89 × 10^−6^
Co		1.51 × 10^−9^		1.51 × 10^−9^		1.24 × 10^−9^		1.24 × 10^−9^
Cr	9.22 × 10^−6^	1.29 × 10^−4^	5.04 × 10^−6^	1.43 × 10^−4^	5.98 × 10^−6^	8.36 × 10^−5^	3.27 × 10^−6^	9.28 × 10^−5^
Ni	6.67 × 10^−6^	6.00 × 10^−10^	1.72 × 10^−7^	6.84 × 10^−6^	5.05 × 10^−6^	4.54 × 10^−10^	1.31 × 10^−7^	5.18 × 10^−6^
Pb	7.13 × 10^−7^	5.36 × 10^−11^	7.70 × 10^−8^	7.90 × 10^−7^	4.91 × 10^−7^	3.69 × 10^−11^	5.31 × 10^−8^	5.44 × 10^−7^
Total	3.65 × 10^−5^	1.30 × 10^−4^	7.94 × 10^−6^	1.74 × 10^−4^	2.99 × 10^−5^	8.44 × 10^−5^	5.90 × 10^−6^	1.20 × 10^−4^

## Data Availability

The data that support the findings of this study are available from the corresponding author on reasonable request.

## Supplementary Materials

The following supporting information can be downloaded at https://www.mdpi.com/article/10.3390/toxics13121008/s1: Table S1: Quality control values of potentially toxic element measurement; Table S2: The values of parameters for non-carcinogenic and carcinogenic risk assessment; Equation (S1): Carcinogenic ingestion rate (IR) calculation; Table S3: The reference values for non-carcinogenic and carcinogenic risk assessment; Table S4: The enrichment factor (EF) of PTEs in indoor household dust across the haze and non-haze seasons in UNT; Table S5: The contamination factor (CF) and pollution load index (PLI) of PTEs in indoor household dust across the haze and non-haze seasons in UNT; Table S6: The index of geo-accumulation (Igeo) of PTEs in indoor household dust across the haze and non-haze seasons in UNT.
