# Supplementary material for: Contamination and Health Risk Assessment of Potentially Toxic Elements in Household Dust Across the Haze Season in Upper Northern Thailand
Source: Toxics. 2025 Nov 21;13(12):1008. doi: 10.3390/toxics13121008 (PMC12737222; doi:10.3390/toxics13121008)
Supplement: Supplementary file 1 [file toxics-13-01008-s001.zip › toxics-3986025-supplementary.pdf]

Supplementary Information

Contamination and Health Risk Assessment of Potentially Toxic Elements in Household Dust Across the Haze Season in Upper Northern Thailand

Table S1 Quality control values of potentially toxic element measurement.

Table S2 The values of parameters for non-carcinogenic and carcinogenic risk assessment.

Equation S1 Carcinogenic ingestion rate (IR) calculation

Table S3 The reference values for non-carcinogenic and carcinogenic risk assessment.

Table S4 The enrichment factor (EF) of PTEs in indoor household dust across the haze and non-haze seasons in UNT.

Table S5 The contamination factor (CF) and pollution load index (PLI) of PTEs in indoor household dust across the haze and non-haze seasons in UNT.

Table S6 The index of geo-accumulation (Igeo) of PTEs in indoor household dust across the haze and non-haze seasons in UNT.

*Corresponding author

Tippawan Prapamontol

E-mail: tippawan.prapamontol@cmu.ac.th

Table S1 Quality control values of potentially toxic element measurement

PTEs	% Detection	LOD (mg kg^{-1})	Standard recovery ± SD (%)	SRM2584			%R.S.D
				Average (mg kg^{-1})	Average (mg kg^{-1})	Average (mg kg^{-1})	
Al	100%	0.84	89.4 ±3.6	21060	23200	90.8	3.33
As	99%	2.08	83.9 ± 3.3	14.56	17.4	83.7	1.35
B	100%	7.24	80.8 ± 3.8	911			0.66
Ba	99%	0.47	97.7 ± 2.2	1052	1300	80.9	0.95
Cd	92%	0.21	93.8 ± 3.3	9.31	10	93.1	0.57
Co	98%	0.42	96.3 ± 2.7	8.22	10	82.2	0.56
Cr	100%	1.11	92.9 ± 4.7	111	135	82.2	0.60
Cu	98%	0.58	97.6 ± 3.2	288	320	90.1	0.81
Mn	100%	0.51	97.0 ± 4.9	299	370	80.7	0.66
Mo	96%	0.6	98.5 ± 4.6	4.93	5.5	89.7	0.08
Ni	100%	1.12	81.9 ± 5.8	73.10	90	81.2	0.77
Pb	99%	0.6	86.8 ± 7.8	8022	9761	82.2	0.91
Sb	97%	0.71	83.6 ± 3.5	12.25	14	87.5	0.31
V	100%	0.43	97.0 ± 4.3	29.45	34	86.6	0.47
Zn	99%	1.54	83.3 ± 5.4	2140	2580	83.0	2.07

Table S2. The values of parameters for non-carcinogenic and carcinogenic risk assessment

Abbrev	Parameter	Value		Unit	Ref
		Adults	Children		
	Non-carcinogenic risk				
ABS	Absorption factor (dermal)	0.001	0.001		US EPA[1]
AT	Averaging time	8,760	2,190	days	Doyi et al., 2019[2]
BW	Body weight	60	15	kg	Somsunun et al. 2022 [3]
CF	Conversion factor	1.00×10^{-6}	1.00×10^{-6}	mg kg^{-1}	Isley et al., 2022[4]
ED	Exposure duration	24	6	years	Hou et al., 2019[5]
EF	Exposure frequency	350	350	days year^{-1}	US EPA[1]
ET	Exposure time	24	24	hours day^{-1}	US EPA[1]
IngR	Ingestion rate^	100	200	mg day^{-1}	US EPA, 2011[6]
InhR	Inhalation rate	16.3	9.3	m^3 day^{-1}	Hou et al., 2019[5]
PEF	Soil to air particulate emission factor	1.36×10^9	1.36×10^9	m^3 kg^{-1}	US EPA[1]
SA	Skin surface area	5,700	2,373	cm^2	US EPA[1]
SL	Solid to skin adherence factor	0.07	0.2	mg cm^{-2}	US EPA[1]
	Carcinogenic risk				
24	Hours per day	24	24	hours	US EPA[1]
ABS	Absorption factor (dermal)	0.001	0.001		Hou et al., 2019[5]
AT	Averaging time	365*70	365*70	days	US EPA[1]
CF	Conversion factor	1.00×10^{-6}	1.00×10^{-6}	mg kg^{-1}	Isley et al., 2022[4]
DFS	Age adjusted soil dermal factor	362.4	362.4	mg year kg^{-1} day^{-1}	Doyi et al., 2019[2]
ED	Exposure duration	26	6	years	US EPA[1]
EF	Exposure frequency	350	350	days year^{-1}	US EPA[1]
ET	Exposure time	24	24	hours day^{-1}	US EPA[1]
IR	Intake rate	113	200	mg year kg^{-1} day^{-1}	US EPA[1]
LT	Lifetime	70	70	years	US EPA[1]
PEF	Soil to air particulate emission factor	1.36×10^9	1.36×10^9	m^3 kg^{-1}	US EPA[1]

Equation S1. Carcinogenic ingestion rate (IR) calculation

$$IR = \left[\frac{(ED_{\text{child}} \times IR_{\text{child}})}{BW_{\text{child}}} + \frac{((ED_{\text{adult}} - ED_{\text{child}}) \times IR_{\text{adult}})}{BW_{\text{adult}}}\right]$$

Table S3 The reference values for non-carcinogenic and carcinogenic risk assessment

Elements	Non-carcinogenic			Carcinogenic		
	RfD_{ing}	RfD_{inh}	RfD_{der}	SF_{ing}	SF_{inh}	SF_{der}
Al	1.00 [a]	5.00×10^{-3} [a]				
As	3.00×10^{-4} [a]	1.50×10^{-5} [a]	1.23×10^{-4} [c]	1.50 a	4.30×10^{-3} [a]	1.50 [a]
B	2.00×10^{-1} [a]	2.00×10^{-2} [a]				
Ba	2.00×10^{-1} [a]	5.00×10^{-4} [a]				
Cd	1.00×10^{-4} [a]	1.00×10^{-5} [a]	1.0×10^{-5} [c]	3.80×10^{-1} [d]	7.05 [d]	15.2 [d]
Co	3.00×10^{-4} [a]	6.00×10^{-6} [a]	1.6×10^{-2} [c]		9.00×10^{-3} [a]	
Cr (VI)	9.00×10^{-4} [a]	3.00×10^{-5} [a]	6.00×10^{-5} [c]	1.60×10^{-1} [a]	42 [d]	20 [c]
Cu	4.00×10^{-2} [a]	4.02×10^{-2} [c]	1.2×10^{-2} [c]			
Mn	2.40×10^{-2} [a]	5.00×10^{-5} [a]	1.84×10^{-3} [c]			
Mo	5.00×10^{-3} [a]	2.00×10^{-3} [a]				
Ni	2.00×10^{-2} [a]	1.00×10^{-5} [a]	5.40×10^{-3} [c]	1.54×10^{-1} [e]	2.60×10^{-4} [a]	9.10×10^{-1} [a]
Pb	3.50×10^{-3} [b]	3.52×10^{-3} [c]	5.25×10^{-4} [c]	8.50×10^{-3} [f]	1.20×10^{-5} [f]	2.10×10^{-1} [a]
Sb	4.00×10^{-4} [a]	3.00×10^{-4} [a]				
V	5.04×10^{-3} [a]	1.00×10^{-4} [a]				
Zn	3.00×10^{-1} [a]	3.00×10^{-1} [a]	6.00×10^{-2} [c]			

a: US EPA (RSL)[1]

b: Ferreira-Baptista and De Miguel., 2005[7]

c: US EPA 2011

c: Doyi et al., 2019[2]

d: Wignall et al., 2018[8]

e: OEHHA[9]

Table S4. The enrichment factor (EF) of PTEs in indoor household dust across the haze and non-haze seasons in UNT.

PTEs	Enrichment factor (EF)										P-value
	Haze season					Non-haze season					
	n	Mean	SD	Median	IQR	n	Mean	SD	Median	IQR	
Al	58	1.00	-	1.00	-	160	1.00	-	1.00	-	1.000
As	54	33.79	42.55	28.71	11.23	152	28.83	19.52	25.35	16.13	0.228
B	54	9.37	4.87	8.28	4.81	154	8.24	6.57	6.99	3.24	0.091
Ba	54	1.78	1.13	1.66	0.48	151	1.62	0.81	1.47	0.63	0.234
Cd	52	86.50	149.30	50.33	67.30	141	88.90	148.77	41.54	56.61	0.528
Co	53	0.74	0.78	0.63	0.43	152	0.57	0.44	0.45	0.33	0.251
Cr	54	2.45	1.80	2.14	1.06	154	1.70	1.01	1.48	0.55	0.000
Cu	52	19.00	11.82	16.66	13.70	151	17.01	14.45	12.64	10.85	0.115
Mn	54	3.51	1.27	3.54	1.82	154	3.41	1.41	3.11	1.65	0.628
Mo	52	5.17	2.40	4.67	2.71	148	3.82	2.20	3.40	1.87	0.000
Ni	54	3.94	2.73	3.30	2.58	154	2.86	1.88	2.47	1.44	0.001
Pb	54	18.28	13.89	14.15	10.35	152	12.78	8.92	10.17	7.35	0.000
Sb	51	108.39	78.75	85.34	84.13	151	120.51	141.04	70.99	57.87	0.673
V	54	1.29	0.29	1.27	0.40	154	1.17	0.22	1.17	0.30	0.004
Zn	53	33.46	20.60	26.52	23.01	152	26.38	19.21	20.79	17.19	0.002

Table S5. The contamination factor (CF) and pollution load index (PLI) of PTEs in indoor household dust across the haze and non-haze seasons in UNT.

PTEs	Contamination Factor (CF) and Pollution Load Index (PLI)										P-value
	Haze season					Non-haze season					
	n	Mean	SD	Median	IQR	n	Mean	SD	Median	IQR	
Al	58	0.22	0.14	0.21	0.08	160	0.24	0.08	0.23	0.09	0.687
As	53	7.37	6.23	5.87	3.87	152	6.84	4.11	5.73	4.22	0.934
B	53	2.21	1.72	1.84	0.71	154	1.90	1.28	1.74	0.65	0.135
Ba	53	0.41	0.28	0.35	0.12	151	0.38	0.16	0.35	0.13	0.655
Cd	51	17.07	23.88	11.27	14.50	141	20.65	35.17	8.67	10.26	0.679
Co	52	0.16	0.16	0.13	0.13	152	0.13	0.10	0.11	0.09	0.333
Cr	53	0.61	0.74	0.49	0.20	154	0.40	0.17	0.36	0.16	0.000
Cu	51	3.98	2.18	3.18	2.24	151	3.93	3.12	2.88	2.08	0.374
Mn	53	0.80	0.45	0.72	0.33	154	0.81	0.35	0.71	0.40	0.750
Mo	51	1.19	0.78	1.00	0.54	148	0.88	0.43	0.78	0.42	0.000
Ni	53	0.87	0.59	0.68	0.50	154	0.66	0.39	0.57	0.31	0.001
Pb	53	4.22	3.49	2.82	2.61	152	2.99	2.02	2.47	1.67	0.012
Sb	51	24.23	16.24	16.61	19.22	151	27.24	28.74	16.61	12.70	0.925
V	53	0.31	0.15	0.30	0.15	154	0.28	0.10	0.28	0.11	0.486
Zn	52	7.68	5.02	5.89	4.50	152	5.89	3.44	5.01	3.16	0.004
PLI	54	0.83	0.43	0.80	0.37	154	0.71	0.18	0.70	0.23	0.004

Table S6. The index of geo-accumulation (Igeo) of PTEs in indoor household dust across the haze and non-haze seasons in UNT.

PTEs	Index of geo-accumulation (Igeo)										P-value
	**Haze season**					**Non-haze season**					
	**n**	**Mean**	**SD**	**Median**	**IQR**	**n**	**Mean**	**SD**	**Median**	**IQR**	
Al	58	-0.76	0.26	-0.80	0.19	160	-0.77	0.19	-0.80	0.16	0.795
As	54	0.62	0.24	0.59	0.30	152	0.60	0.22	0.58	0.30	0.788
B	54	0.10	0.21	0.08	0.18	154	0.06	0.17	0.06	0.17	0.178
Ba	54	-0.61	0.18	-0.63	0.15	151	-0.63	0.14	-0.63	0.16	0.623
Cd	52	0.89	0.35	0.88	0.49	141	0.86	0.43	0.76	0.45	0.617
Co	53	-1.09	0.31	-1.08	0.47	152	-1.13	0.26	-1.12	0.34	0.328
Cr	54	-0.48	0.23	-0.48	0.19	154	-0.61	0.16	-0.62	0.19	0.000
Cu	52	0.37	0.21	0.33	0.26	151	0.33	0.25	0.28	0.29	0.358
Mn	53	-0.31	0.18	-0.32	0.20	154	-0.30	0.18	-0.33	0.23	0.750
Mo	52	-0.15	0.19	-0.17	0.22	148	-0.27	0.18	-0.29	0.23	0.000
Ni	54	-0.30	0.22	-0.35	0.29	154	-0.41	0.20	-0.42	0.23	0.001
Pb	54	0.36	0.29	0.28	0.39	152	0.24	0.22	0.22	0.28	0.007
Sb	51	1.12	0.27	1.04	0.38	151	1.12	0.32	1.04	0.30	0.925
V	54	-0.72	0.18	-0.69	0.23	154	-0.74	0.14	-0.74	0.17	0.407
Zn	53	0.64	0.24	0.59	0.31	152	0.53	0.22	0.52	0.27	0.006

References

1 EPA, U. *US EPA Regional Screening Levels (RSL) Calculator*, <https://epa-prgs.ornl.gov/cgi-bin/chemicals/csl_search> (

2 Doyi, I. N. Y., Isley, C. F., Soltani, N. S. & Taylor, M. P. Human exposure and risk associated with trace element concentrations in indoor dust from Australian homes. *Environment International* **133**, 105125, doi:https://doi.org/10.1016/j.envint.2019.105125 (2019).

3 Somsunun, K. *et al.* Estimation of lung cancer deaths attributable to indoor radon exposure in upper northern Thailand. *Sci Rep* **12**, 5169, doi:10.1038/s41598-022-09122-y (2022).

4 Isley, C. F. *et al.* International Analysis of Sources and Human Health Risk Associated with Trace Metal Contaminants in Residential Indoor Dust. *Environ Sci Technol* **56**, 1053-1068, doi:10.1021/acs.est.1c04494 (2022).

5 Hou, S. *et al.* Pollution characteristics, sources, and health risk assessment of human exposure to Cu, Zn, Cd and Pb pollution in urban street dust across China between 2009 and 2019. *Environment International* **128**, 430-437, doi:https://doi.org/10.1016/j.envint.2019.04.046 (2019).

6 US EPA. (U.S. Environmental Protection Agency, Washington, DC, 2011).

7 Ferreira-Baptista, L. & De Miguel, E. Geochemistry and risk assessment of street dust in Luanda, Angola: A tropical urban environment. *Atmospheric Environment* **39**, 4501-4512, doi:https://doi.org/10.1016/j.atmosenv.2005.03.026 (2005).

8 Wignall, J. A. *et al.* Conditional Toxicity Value (CTV) Predictor: An <i>In Silico</i> Approach for Generating Quantitative Risk Estimates for Chemicals. *Environmental Health Perspectives* **126**, 057008, doi:doi:10.1289/EHP2998 (2018).

9 Office of Environmental Health Hazard Assessment (OEHHA). *Technical Support Document for Cancer Potency Factors 2009*, <https://oehha.ca.gov/air/crnr/technical-support-document-cancer-potency-factors-2009> (
